# Neural Network for Principle of Least Action

**DOI:** 10.1021/acs.jcim.2c00515

**Published:** 2022-07-05

**Authors:** Beibei Wang, Shane Jackson, Aiichiro Nakano, Ken-ichi Nomura, Priya Vashishta, Rajiv Kalia, Mark Stevens

**Affiliations:** † Collaboratory for Advanced Computing and Simulations, University of Southern California, Los Angeles, California 90089, United States; ‡ Center for Integrated Nanotechnologies, Sandia National Laboratory, Albuquerque, New Mexico 87185, United States

## Abstract

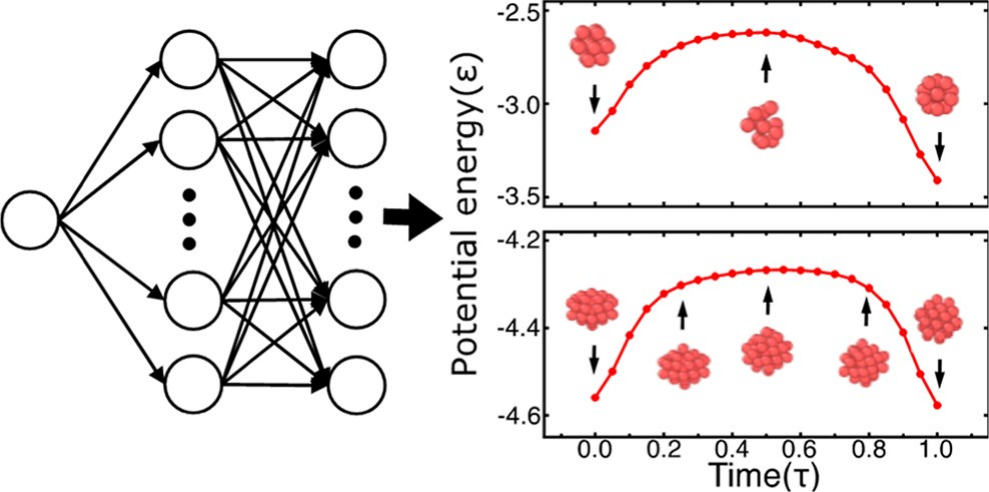

The principle of least action is the cornerstone of classical mechanics, theory of relativity, quantum mechanics, and thermodynamics. Here, we describe how a neural network (NN) learns to find the trajectory for a Lennard-Jones (LJ) system that maintains balance in minimizing the Onsager–Machlup (OM) action and maintaining the energy conservation. The phase-space trajectory thus calculated is in excellent agreement with the corresponding results from the “ground-truth” molecular dynamics (MD) simulation. Furthermore, we show that the NN can easily find structural transformation pathways for LJ clusters, for example, the basin-hopping transformation of an LJ_38_ from an incomplete Mackay icosahedron to a truncated face-centered cubic octahedron. Unlike MD, the NN computes atomic trajectories over the entire temporal domain in one fell swoop, and the NN time step is a factor of 20 larger than the MD time step. The NN approach to OM action is quite general and can be adapted to model morphometrics in a variety of applications.

## 1 Introduction

The principle of least action is a foundational law of physics. It pervades classical mechanics,^1^ theory of relativity,^2^ quantum mechanics,^3^ and thermodynamics.^4^ The action is defined as
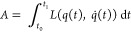
1where *L* is the Lagrangian in classical mechanics and *q*(*t*) and *q̇*(*t*) are the system trajectory and its time derivative, respectively. Equation 1 embodies a boundary-value problem in which *A* is minimized with respect to *q*(*t*), subject to the constraints imposed by the initial and final configurations at time *t*_0_ and *t*_1_, respectively.^5^ The differential equations arising from the principle of least action are the Euler–Lagrange equations, which constitute an initial-value problem. These equations are at the core of molecular dynamics (MD), the preeminent simulation approach in physics, chemistry, materials science, and biology.^6^ The essential input to MD is interatomic potential energy from which forces are calculated and the equations of motion are integrated over discretized time with a finite-difference scheme.^7^ The output is phase-space trajectories {*q*(*t*), *q̇*(*t*)} from which structural, mechanical, thermodynamic, and dynamical properties of the system can be computed and compared with experimental measurements.

In recent years, there has been a surge of interest in applying machine learning (ML) tools in MD tasks. Atomic force fields have been developed using neural networks trained by data from quantum-mechanical calculations.^8,9^ Predictive models based on kernel ridge regression, support vector machine, random forest, and other techniques have been employed to predict material properties such as band gaps,^10^ elastic constants,^11^ dielectric constants, and thermoelectric properties.^12−14^ For example, Bayesian optimization methods have been used to discover optimal layered materials for targeted properties such as electronic band structure and thermal-transport coefficients.^15^ On the other hand, original ML models have been built to learn the basic laws of physics for simple systems such as a mass-spring, a double pendulum, and even the case of light refraction.^16−18^

In this paper, we explore the application of ML in a different context, that is, how a NN learns the principle of least action and provides atomic trajectories by minimizing the Onsager–Machlup (OM) action^19^
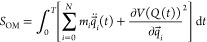
2where *m*_*i*_ is the mass and  is the second-order time derivative of the position vector *q⃗*_*i*_(*t*) of the *i*th particle. In eq 2, *Q*(*t*) collectively represents the coordinates of all the particles in the system at time *t* and *V*(*Q*(*t*)) is the potential energy of the system. The genesis of the OM action was Onsager’s seminal papers in which he developed reciprocal relations for irreversible transport process and proposed that the probability of paths of a diffusion process is exponentially small with the exponent proportional to the time integral of dissipation function of the history.^20−22^ Later on, Onsager and Machlup showed that minimization of eq 2 would produce the most probable trajectory.^20^ Action formulation is a popular optimization approach to atomistic boundary-value problems since a global minimum is guaranteed.^23^ A straightforward minimization of the discretized OM action involves second-order derivatives which are computationally expensive to calculate for large systems. Passerone and Parrinello add the energy-conservation constraint to the OM action and minimize the resulting function by making the following transformation
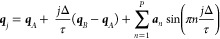
3which automatically satisfies the boundary condition. The minimization is performed with respect to Fourier coefficients ***a***_*n*_ to get atomic trajectories. It has been pointed out that these solutions are strongly dependent upon the initial values of ***a***_*n*_.^24^

Here, we demonstrate how a NN optimizes the OM action (eq 2) for a system of Lennard-Jones (LJ) atoms^25^ and produces atomic trajectories in one fell swoop over the entire time domain between *t*_0_ and *t*_1_. The atomic trajectories generated by the NN are in excellent agreement with the “ground-truth” MD trajectories even though the time step taken by the NN is 20 times larger than the MD time step. We also demonstrate that the NN can easily find transformation pathways and energy barriers between different structures of LJ clusters. The NN-based approach to OM action can be easily implemented in any statistical ensemble, and it is straightforward to include constraints, invariances, and conservation laws in the NN loss function.

## 2 Methods

**Figure 1:**
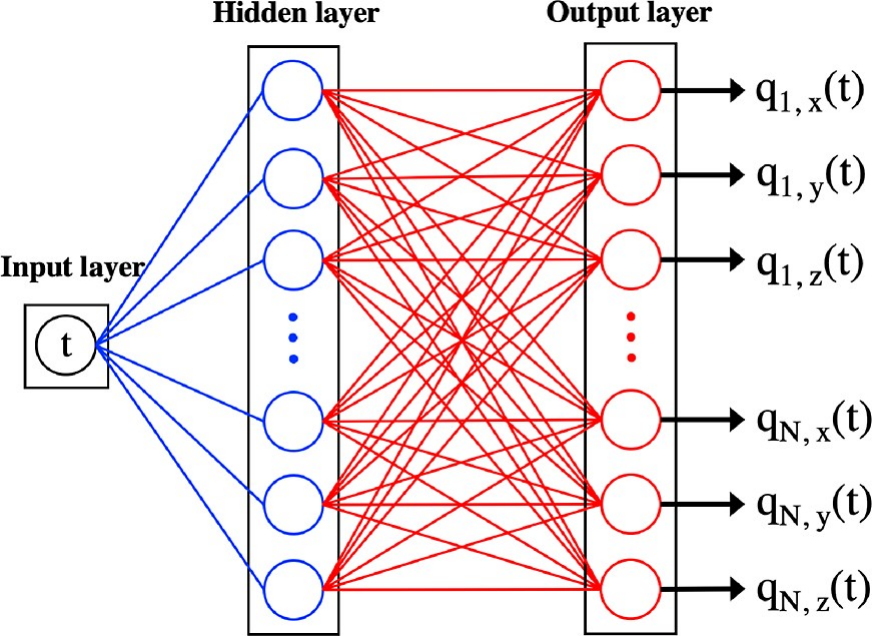
NN consists of an input layer, a hidden layer (blue), and an output layer (red), which gives the atomic coordinates whose first and second derivatives provide velocity and acceleration, respectively. The blue and red edges represent that the weights and each node has a bias.

Figure 1 shows our NN architecture. It consists of an input node, a hidden layer with *n* units, and an output layer with *d*×*N* units, where *N* is the number of atoms and *d* is the dimensionality of the system. The network outputs the trajectories of all the atoms in the system, that is, the cartesian components {*q*_*i*,*x*_, *q*_*i*,*y*_, and *q*_*i*,*z*_} from *i* = 1 to *N*. The system configuration for all spatial degrees of freedom is calculated by feeding time into the input layer and letting it propagate through the hidden layer to the output layer. More details of the model output can be found in the Supporting Information.

We evaluate the OM action numerically by discretizing the time integral in eq 3 into *N*_*t*_ grid points. The time increment is Δ*t* = τ/*N*_*t*_, where τ is the time unit computed from the chosen LJ interaction for liquid Argon.^26^ The loss function *L* for the NN includes not only the OM action but also constraints for boundary conditions and energy conservation
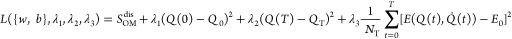
4
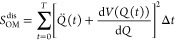
5
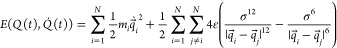
6where {*w*, *b*} represents all the weights and biases in the NN. For simplicity, we use *Q*(*t*) to denote the model output *Q*({*w*, *b*}, *t*). *Q*_0_ and *Q*_T_ respectively represent initial and final coordinates of all the atoms in the system. λ’s are hyper-parameters, which are chosen using random search schema for balancing the magnitude of individual terms (details provided in Supporting Information). Note that the last term in eq 4 is the energy-conservation constraint, where *E*_0_ is the total energy of the system. In eq 6, *E*(*Q*(*t*),*Q̇*(*t*)) is the total energy, and ε and σ are the parameters for the LJ potential.

We minimize the loss function in eq 4 using the Nestrov-and-Adam (NADAM) optimizer.^27^ The gradient of the loss function is calculated numerically using Google’s JAX library.^28^ After training the network, we compute atomic trajectories and compare them with the “ground-truth” MD simulations performed using the same set of initial conditions. We have trained the network to predict trajectories of liquid Argon systems consisting of up to 500 atoms in three dimensions. We have also used the NN to find transition pathways and energy barriers between different structures of LJ clusters. More details about the NN model and the atomic systems we study are given in the Supporting Information.

## 3 Results

The NN learns to compute atomic trajectories of two- and three-dimensional LJ systems from the OM least action principle. Here, we present results for a 3D system containing 500 atoms, which was trained on a NN with 125 neurons in the hidden layer. Results in this section are generated on *N*_t_ = 25 time increments, which correspond to a time step (∼40 fs) that is 20 times the MD timestep (2 fs). It is worth noting that the NN is designed to approach the most probable phase-space trajectory for a system given initial and final configurations, rather than outperform MD at the computing speed of atomic trajectories.

**Figure 2:**
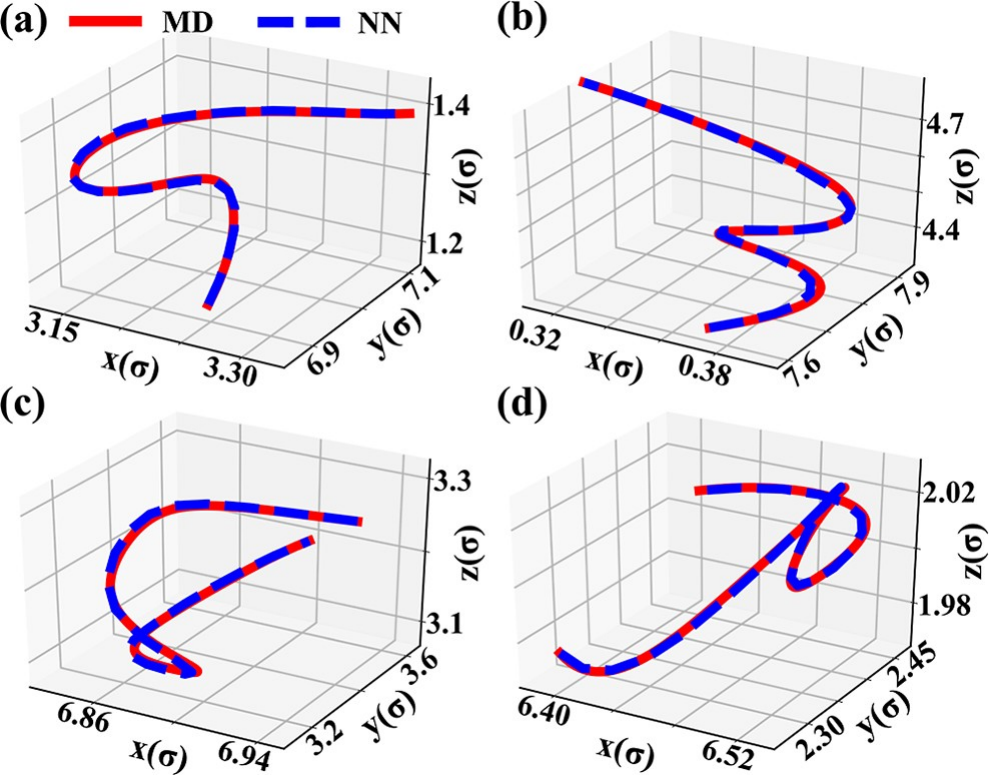
Visualization of atomic trajectories in the 500-atom system. NN (solid blue) and MD (solid red) trajectories show that the NN can reproduce non-trivial paths over a time period of 1 ps.

Figure 2 shows a comparison of NN and MD trajectories for four randomly chosen particles in the 500-atom LJ system over a time period of 1 ps. These trajectories cover a time scale beyond the straight-line ballistic motion and possess sharp changes in direction. It is remarkable that the NN is able to reproduce the dynamics at the individual particle level. From this level of matching for particle trajectories, it is not surprising that the NN results for all other quantities are in excellent agreement with the “ground truth” MD results.

**Figure 3:**
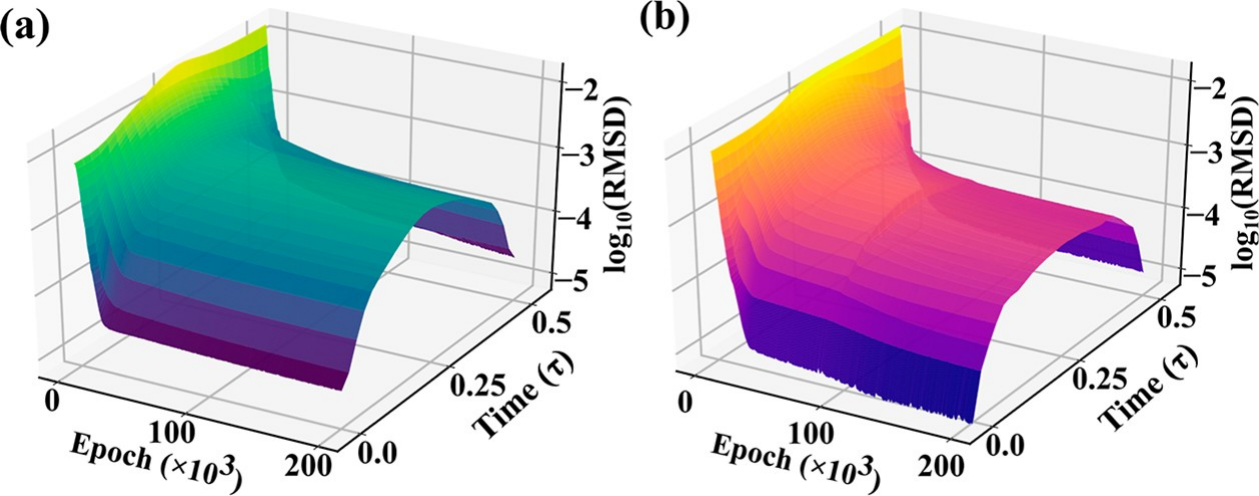
Root-mean-square deviation (RMSD) between MD and NN trajectories (a) Shows the deviation between MD and NN trajectories for a 256-atom LJ system. (b) figure shows how the RMSD between NN and MD trajectories for a 500-particle system decreases with the number of epochs and changes with time. The boundary-condition constraints in the loss function ensure that the RMSDs are much smaller near the initial and final configurations than in the middle of the time domain. Here, τ = 2 ps.

Figure 3 shows the root mean-square deviations (RMSDs) between the NN and MD trajectories over the entire time domain as a function of epochs for 256- and 500-atom LJ systems. The RMSDs for positions *Q* are indeed very small, resulting in excellent matching of the NN and MD trajectories. The RMSDs between the NN and MD trajectories are negligible near the initial and final configurations because of boundary-condition constraints in the loss function. Even in the middle of the time domain, the RMSD does not exceed 10^–4^. The difference between the potential energies computed by the NN and MD is therefore negligible. The behavior of the RMSDs for momenta *P* as a function of epoch is similar to the RMSDs of trajectories, but the deviations are larger for the following reasons: first, referring to eqs 4–6, the time derivatives are described with fewer parameters than the positions as the biases outside the activation function are killed by the derivative; second, all quantities in the loss function depend explicitly on *Q* and only two depend on *P*. Since we have a finite number of parameters, they can only approximate the function up to some error. These errors magnify as we take higher derivatives.

**Figure 4:**
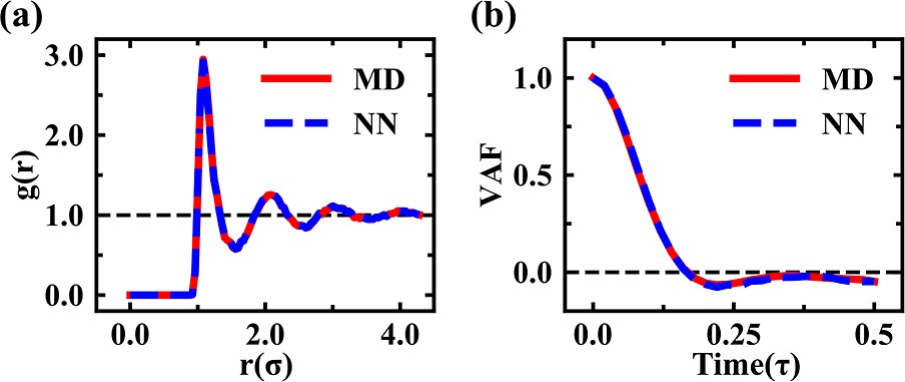
Comparison between the NN and MD simulation results. (a) Radial distribution functions and (b) velocity autocorrelation functions from MD (red) and NN (blue) dynamics.

In Figure 4, we compare the results from the NN and MD for some standard simulation quantities. For example, the structure of the liquid is characterized by the radial distribution function^29^*g*(*r*) shown in Figure 4a. Slightly below *r* = 1, *g*(*r*) = 0 because the particles cannot overlap and the NN trajectory maintains this feature. The peaks in *g*(*r*) corresponding to the nearest and second nearest neighbors indicate that our model is able to obtain the correct liquid structure. To characterize the particle dynamics, we calculated the velocity autocorrelation function^30^ (VAF) shown in Figure 4b. The match between NN and MD is good, and the correlation time *t*_c_, when VAF = 0 for the first time, is about 0.16 in the unit of τ = 2 ps. Beyond *t*_c_ the velocities and, in general, the particle dynamics are not correlated with the initial dynamics of the particles. This quantity is important in calculating time averages, as a time series of data with points separated by at least *t*_c_ are not correlated and contribute independently to the average quantity.

We have also used our NN to find transition pathways between different structures of LJ clusters. The number of conformations of a cluster can be very large,^31^ and the potential energy surface (PES) of a cluster has a large number of local minima in which it can get trapped, making it very difficult to identify the global minimum.^32^ Finding the global minimum of a cluster or protein is considered an NP-hard problem;^33^ that is, the global minimum cannot be found in polynomial time.

There have been numerous computational studies of LJ clusters consisting of tens to hundreds of atoms. Global minima of almost all the clusters up to LJ_150_ atoms have been found. Most of them have Mackay icosahedral structures as their global minima.^34^ However, there are a few exceptions, namely, LJ_38_, LJ_75–77_, and LJ_102–104_.^35^ The global minimum of LJ_38_ is a face centered cubic (FCC) truncated octahedron, and the global minima of LJ_75–77_ and LJ_102–104_ are Marks dodecahedra.^35^

**Figure 5:**
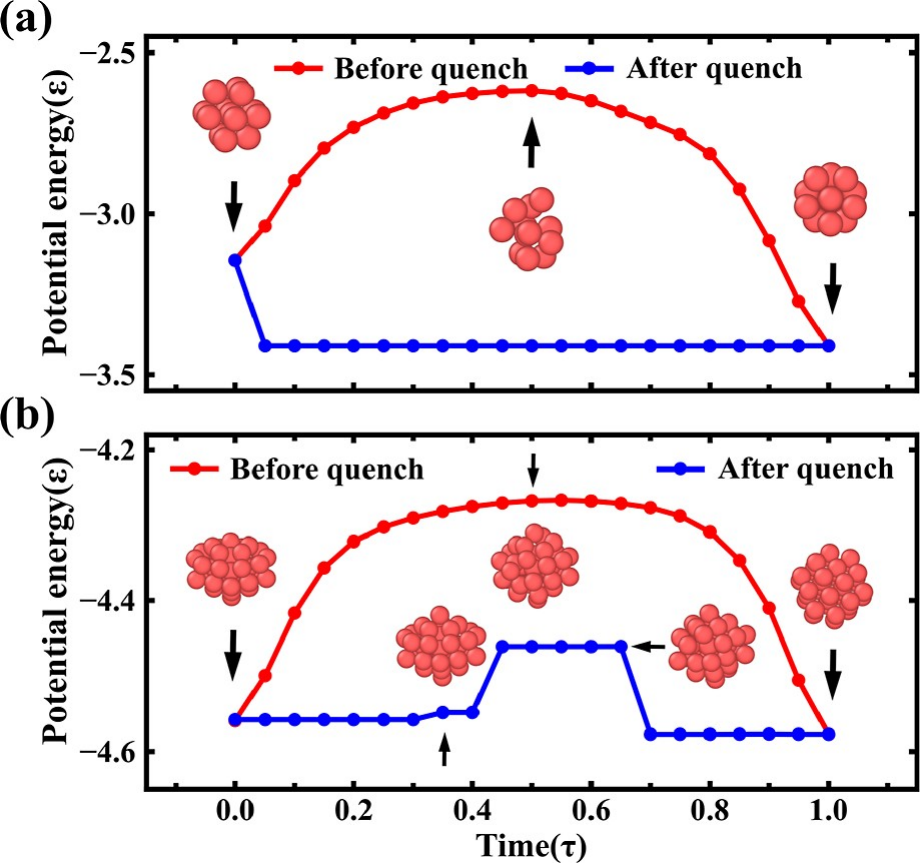
Panels (a) and (b) show potential energies per atom for LJ_13_ and LJ_38_ clusters, respectively, as a function of time. The intermediate structures of the clusters are also shown in panels (a) and (b) red curves are the NN potential energies and the blue curves are the PES obtained by quenching NN configurations. LJ_13_ quickly quenches into a Mackay icosahedra global minimum. The LJ_38_ structure indicates that the cluster remains an incomplete Mackay icosahedron for nearly 0.8 ps. In the next 0.6 ps, the cluster crosses an energy barrier and transforms into an FCC truncated octahedron, which is the global minimum. The LJ_38_ structures just before, during, and after the transition are shown in (b).

Figure 5 shows the structural transition pathway of an LJ_38_ from a local to the global minimum on the PES. For comparison, we have also trained our NN to find the transition of an LJ_13_ to the Mackay icosahedron global minimum. The structural change in LJ_13_ from a truncated FCC to an icosahedron can be easily spotted within a thousand MD steps. The NN finds this transition within a few time steps as shown in Figure 5a. In the case of an LJ_38_, we used the NN to find the transition pathway between the second lowest and the global minimum, that is, from an incomplete Mackay icosahedron to an FCC truncated octahedron. This transition is hard to find by MD simulation, whereas the NN rapidly finds not only the transition pathway but also the potential energy barrier and intermediate conformations between the Mackay icosahedron and the FCC truncated octahedron (see Figure 5b).

To quantify the difference between the transition pathways of LJ_13_ and LJ_38_, we compute the Euclidean distance of the initial and final configurations *Q*_i_ and *Q*_f_. The distance between the two structures is the minimal arc length traveled by a cluster as it transitions from *Q*_i_ to *Q*_f_ in a time period Τ. The minimal distance is defined by a transformation *Q̅*(*t*), which is a 3N-dimensional vector parameterized by time *t*. The infinitesimal distance traveled during the transformation is , and the minimal distance between *Q*_i_ and *Q*_f_ is given by^36^
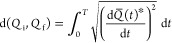
7where *Q̅*(*t*)* is the minimal path determined by the NN. We find that the Euclidean distances between the initial and final configurations of LJ_13_ and LJ_38_ are 0.9 σ and 10.4 σ, respectively. It has been suggested that the global minimum of an LJ_13_ can be located much more easily than that of an LJ_38_ because the former has a single funnel energy landscape and the latter has double funnels leading to the global minimum.^37^

## 4 Conclusions

In this work, we have shown that a NN can correctly generate atomic trajectories of a LJ system by minimizing the OM action. The NN phase-space trajectories, potential and kinetic energies, radial distribution function, and velocity autocorrelation function are in excellent agreement with the corresponding results from the “ground-truth” MD simulation. We have also demonstrated that the NN can easily find transition pathways and energy barriers between different configurations of typical LJ clusters. Despite the limitation of our method in computing speed, the NN approach to principle of least action has some advantages over the traditional MD method: the NN solves boundary condition problems while the MD cannot, and the NN provides the entire trajectory in one fell swoop with a time step much larger than that of the MD, while maintaining energy conservation over the entire time domain. Our NN approach is generally applicable to systems with complex interatomic interactions other than LJ potential. The encouraging results indicate the possibility of making the NN method a candidate for modeling experimental data. For example, Hills et al. have developed an algorithm based on the least action principle to predict the dynamics of physical systems using observed data.^38^ Our approach combined with theirs can model observational data and predict the dynamics beyond the experimental measurements of systems more than LJ clusters.

## Data Availability

The code and data that support the findings of this study are provided in the Supporting Information; they are also available from the corresponding author upon request.

## Supporting Information Available

Details of the LJ interaction for the system, model pre-training protocol, scaling curves for the time performance and RMSD with respect to the system size, comparisons between MD and NN trajectories with correlation functions for systems having different number of atoms, and transition pathway generated by the NN model for a surface-hopping event in a small 2D LJ cluster (PDF)
