# Supplementary material for: Neural Network for Principle of Least Action
Source: J Chem Inf Model. 2022 Jul 5;62(14):3346–51. doi: 10.1021/acs.jcim.2c00515 (PMC9326973; doi:10.1021/acs.jcim.2c00515)
Supplement: Supplementary file 1 — ci2c00515_si_001.pdf [file ci2c00515_si_001.pdf]

# Supporting Information

# A Neural Network for Principle of Least Action

Beibei Wang*[1], Shane Jackson[1], Aiichiro Nakano[1], Ken-ichi Nomura[1], Priya Vashishta[1], Rajiv Kalia[1], Mark Stevens[2]

[1]Collaboratory for Advanced Computing and Simulations, University of Southern California, Los Angeles, CA 90089

[2]Center for Integrated Nanotechnologies, Sandia National Laboratory, Albuquerque, NM 87185

**Corresponding Author:**

*Beibei Wang: beibeiw@usc.edu

## Lennard-Jones System

Our neural network (NN) for the principle of least action is tested against the "ground truth" molecular dynamics (MD) simulations of a system in which atoms interact via Lennard-Jones (LJ) potential. In reduced units, the pair-wise LJ interaction is given by:

$$V_{ij} = 4\left(\frac{1}{\|\vec{q}_i - \vec{q}_j\|^{12}} - \frac{1}{\|\vec{q}_i - \vec{q}_j\|^{6}}\right) \tag{S1}$$

The interaction parameters for the LJ potential in this work are given in table S1[1]. Molecular dynamics simulation was initiated from the face-centered cubic (FCC) structure. The reduced number density of the system is 0.787 and the reduced time unit $\tau = 2.068$ ps. Periodic boundary conditions were imposed, and equations of motion were integrated with the velocity-Verlet algorithm using a timestep of 2 fs. The system was equilibrated for 2 ns at a reduced temperature of 0.695. We use the coordinates of the particles in the equilibrated system as the initial configuration for the NN simulation. To obtain a final state for our boundary-value problem, we run MD simulations using the same starting state.

| Simulation Parameter | Value |
|---|---|
| $\epsilon/k_b(K)$ | 125.7 |
| σ (nm) | 0.3345 |
| m (a.m.u) | 39.948 |
| Lattice Constant (nm) | 0.575 |

Table S1: Lennard Jones interaction parameters and lattice constant.

## Model details

As shown in Figure 1 in the main text, the architecture of the NN model is a feedforward single-hidden-layer structure with one input and m output units. The output of the network can be written as:

$$Q_m(t, \{w, b\}) = \sum_{i}^{n} w_i^1 g\left(w_i^0 t + b_i^0\right) + b_i^1 \tag{S1}$$

where the superscript "0" labels the parameters in hidden layer and "1" is for the parameters in output layer. The activation function $g{:}\boldsymbol{R}^n \mapsto \boldsymbol{R}^n$ is only applied to hidden units. We use this network to predict the trajectory from coupled Newton's PDEs for many particles. The output dimension m equals the simulation dimensionality multiplied by the total number of particles. In the case of 16 particle and 3-dimensional simulation, out output size is 48, i.e., given the boundary conditions and the discretized time grid points, we can produce the trajectories of all 16 particles simultaneously using the NN model. Note that the hyper-parameters $\lambda_1, \lambda_2, \lambda_3$ mentioned in the main text (Eq. 3) control the relative magnitude of each term in the loss function. They are tuned with random search based on model performance, i.e. the RMSD between the recovered intermediate states from NN and the MD results. In this experiment, the scope of each hyperparameter is $(1, 10, 20, \ldots, 100)$ .We finally chose the parameter set ( $\lambda_1, \lambda_2, \lambda_3) = (20, 20, 10)$ . The code and data used in this work are provided online and available upon request.*

We have studied how the proposed NN model scales with the number of units in the hidden layer and discretization of the time domain on an Intel(R) Xeon(R) CPU E5-2640 machine with 2.60 GHz clock speed. Figure S1(a) shows how the training run time scales with the system size. The behavior appears quadratic because the run time is dominated by the LJ force calculation. The linked-list methd[2] will be employed to reduce the scaling of the run time with the system size to O($N$) for future work. Surprisingly, with the help of Google JAX, figure S1 (b) indicates that increasing the number of neurons in the hidden layer doesn't affect the run time appreciably: it remains ~210 seconds for 1,000 epochs of training. However, changing the grid size of the time domain increases the computing time significantly as shown in figure S1(c), optimization of this part will be developed in future work.

* https://github.com/Ben-USC/Neural_Network_x_OM_Action

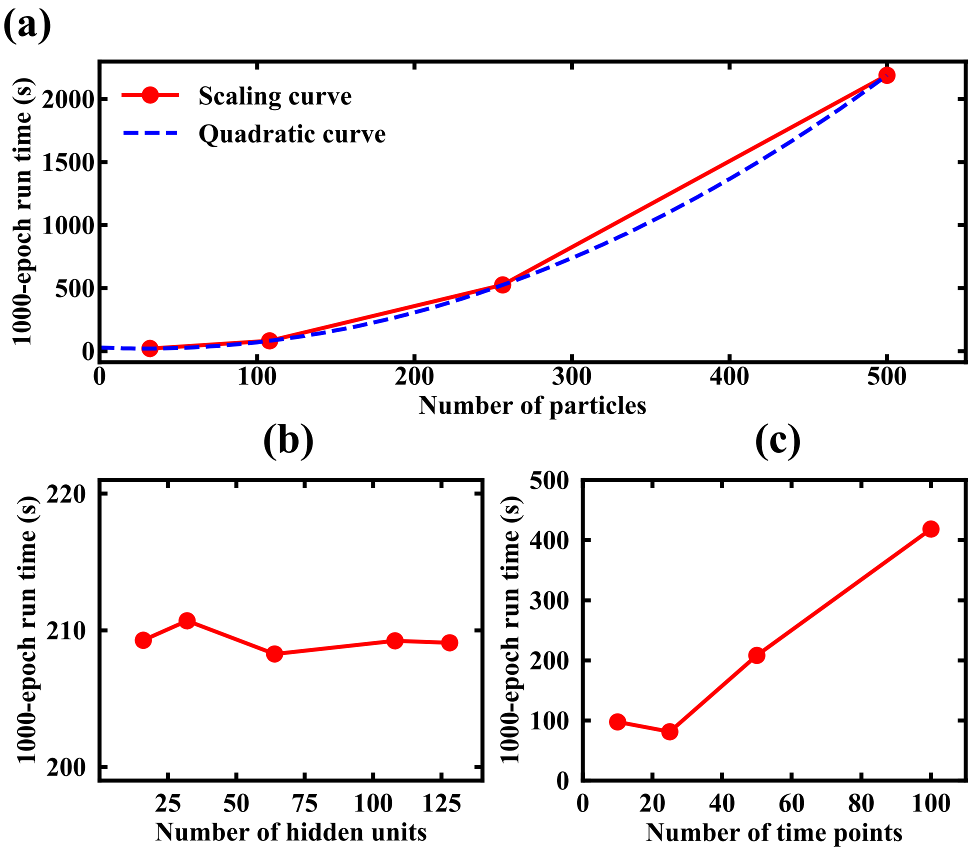


**Figure S1:** Run time scaling of NN for 108-particle system. (a), (b) and (c) are the run time for every 1000 training epoch as a function of the number of particles in the system, number of units in the hidden layer and the size of the time grid, respectively.

Figure S2 shows how the RMSD scales with respect to the size of the hidden layer and the time grid for a 108-atom system in our study. This figure could be misleading if not treated carefully. Figure S2(a) shows that, increasing the number of hidden neurons is not always beneficial for reducing RMSD of the model due to the potential risk of overfitting. However, the reason for the performance improvement with more hidden neurons (> 100) and time points (>100) remains to be explored.

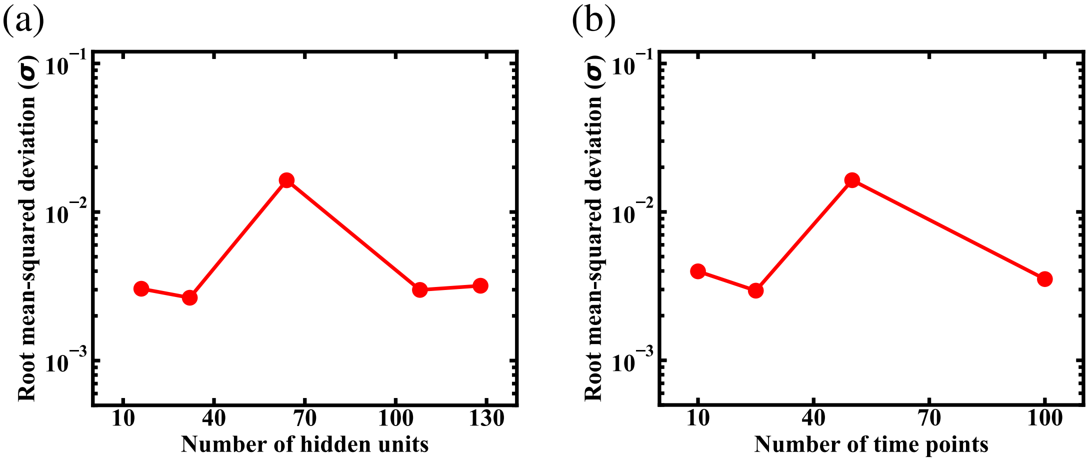


**Figure S2:** Error scaling of NN for 108-particle system. (a) and (b) are the RMSD of the model output as a function of the number of hidden units and the size of the time grid, respectively.

Figure S3 shows how the RMSD scales with different number of particles in the system, where we consider 32, 64, 108 and 500-atom systems and an NN architecture with 64 units in the hidden layer. RMSD of the 500-particle system is approximately 1 order of magnitude higher than that of 100-particle system.

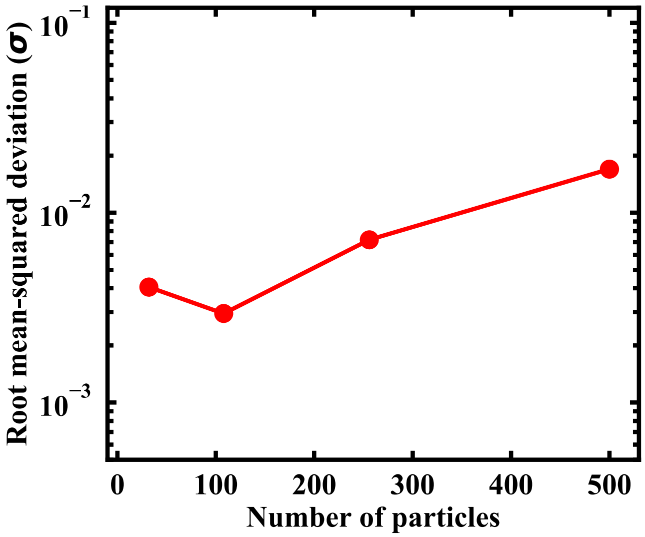


**Figure S3:** RMSD scaling of NN with respect to the number of particles in the system.

## Pre-training

Overlapping of particle trajectories will cause large amount of loss due to LJ potential. To avoid this at the parameter initialization stage, we have employed a pre-training strategy as

follows: we first randomize the parameters $\{w, b\}$ using Gaussian distribution, then with the knowledge of the initial and final state of the system, we pre-train the network using the following loss function:

$$L^{pre}(\{w,b\}) = \left(\vec{Q}(\{w,b\},0) - \vec{q}_0\right)^2 + \left(\vec{Q}(\{w,b\},T) - \vec{q}_T\right)^2 + \sum_{i=0}^{T/2}\left(\dot{\vec{Q}}(\{w,b\},i) - \vec{v}_0\right)^2 + \sum_{j=T/2+\Delta t}^{T}\left(\dot{\vec{Q}}(\{w,b\},j) - \vec{v}_T\right)^2 \qquad \text{(S2)}$$

where $\vec{q}_0$ and $\vec{q}_T$ collectively represent the initial and final positions of atoms, and $\vec{v}_0$ and $\vec{v}_T$ are initial and final velocities of atoms in the system, respectively. Note that the first two terms are constraints on initial and final configurations of the system, while the last two terms provide an estimate of trajectories by making particles move at different but constant velocities for the first and second half of the trajectories.

Although this pre-training method has led to interesting results as presented in the main text, there is still a chance that particles may get too close or even cross their trajectories after pre-training, thus give rise to a tremendous energy loss at the beginning of the main training iteration. One way to avoid this during the pre-training is to add constraints to limit the minimum inter-particle distance in Eq. S2, which will be discussed in future work.

**Supplementary results**

In addition to the 500-atom LJ system presented in the main text, we studied systems with 108 and 256 atoms. In the next two figures, we compare NN results against MD simulations for 256 atoms.

Figure S4 visualizes four randomly chosen trajectories generated by the NN (blue), which are coincident with the "ground-truth" MD trajectories (red). The averaged root-mean-square error between the NN and MD trajectories is on the order of $10^{-4}$ in LJ units.

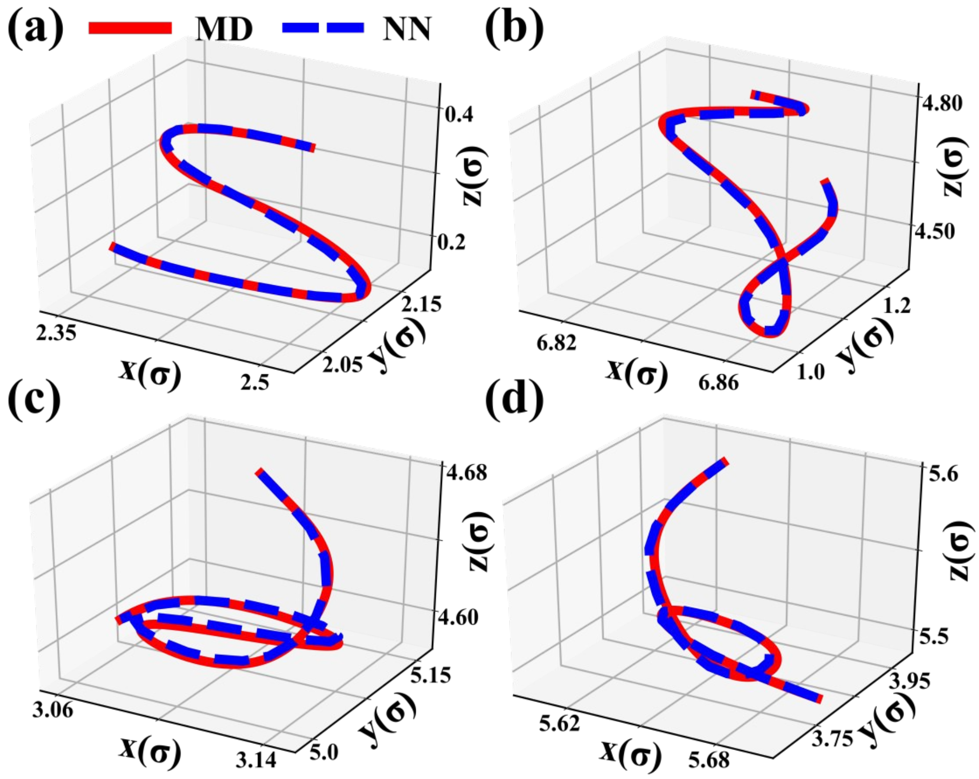


**Figure S4:** NN and MD trajectories for four randomly selected atoms in a system of 256 LJ atoms. Atomic trajectories computed by the NN (blue) are nearly coincident with MD trajectories (red).

Figure S5 compares the NN and MD results for structural and dynamical correlations in the 256-atom system. (a) and (b) show that the NN results for radial distribution function (g(r)) and velocity auto-correlation function (VAF) are in excellent agreement with the corresponding MD results. (c) shows the relative deviation between MD energies and those calculated from the NN trajectories. The relative error is within 0.6 %.

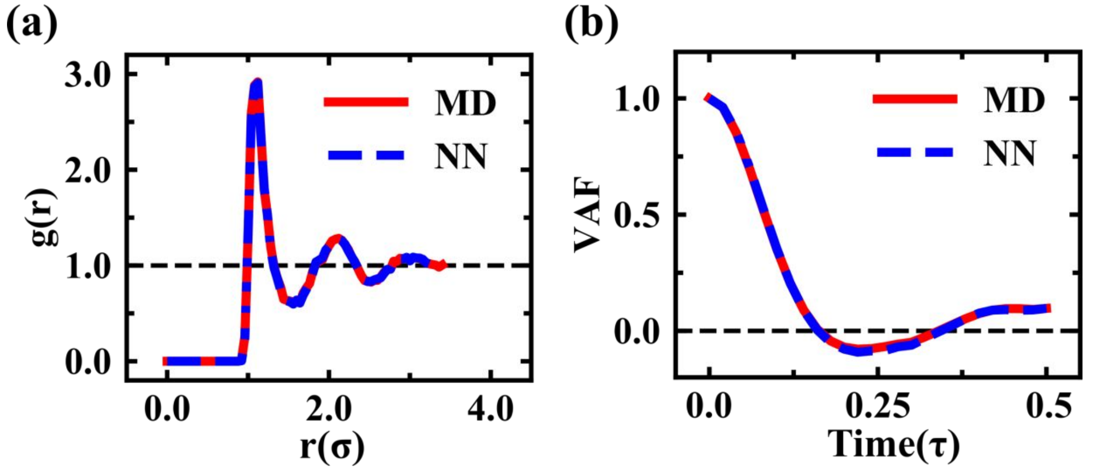


**Figure S5:** (a) radial distribution function and (b) velocity auto-correlation function show the near-perfect agreement between the NN and MD for a 256-atom system.

Figures S6 and S7 show the same degree of agreement between NN and MD results in atomic dynamical trajectories, energies and structures for the 108-atom system.

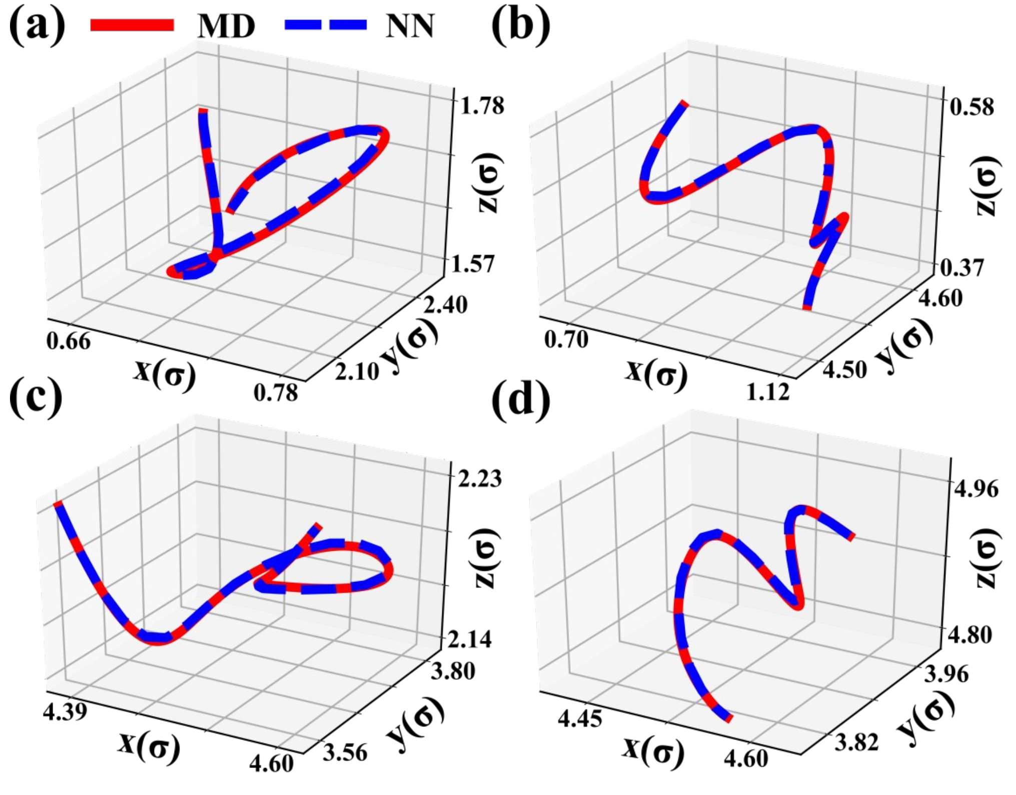


**Figure S6:** Comparison between MD and NN trajectories for four particles selected randomly from the 108-atom system. Red lines are MD trajectories and Blue dots are trajectories computed by the NN.

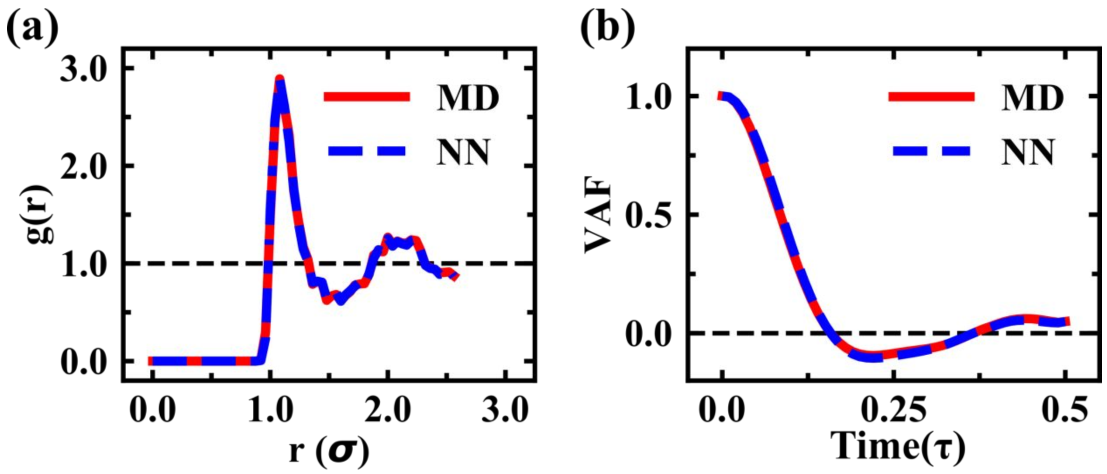


**Figure S7:** (a) radial distribution function and (b) velocity auto-correlation function show the near-perfect agreement between the NN and MD for a 256-atom system.

Figure S8 shows how the root-mean-square deviation between the MD and NN trajectories for the 108-atom system changes with number of epochs and simulation time.

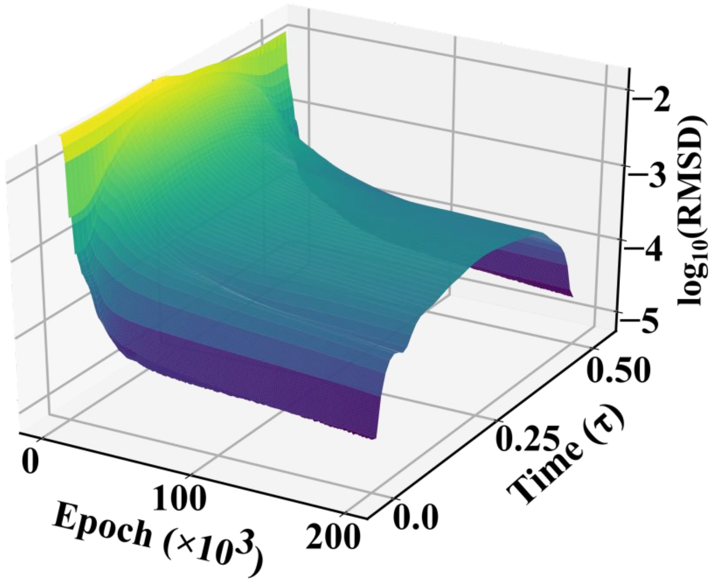


**Figure S8:** Root-mean-square deviation (RMSD) between the MD and NN trajectories for a 108-atom LJ system. The deviation of the NN trajectory from MD decreases with the number of epochs and changes with time. The boundary constraints in the loss function ensure that the error is much smaller near the initial and final configurations than in the middle of the time domain. The magnitude of the deviation is below $10^{-4}$ in LJ units.

As a simple example of another application of our method, we consider a process in which the center atom in a 2D LJ cluster moves to the surface.[3,4] Figure S9(a) shows the transformation

pathway generated by the NN from the initial to the final state. The cluster goes through some intermediate states, which agree with the sampled results from the references mentioned above. S8(b) shows the total and potential energy profiles of the system as a function of time. The standard deviation of the total energy is 0.007 in LJ units, less than 0.6% of the energy value.

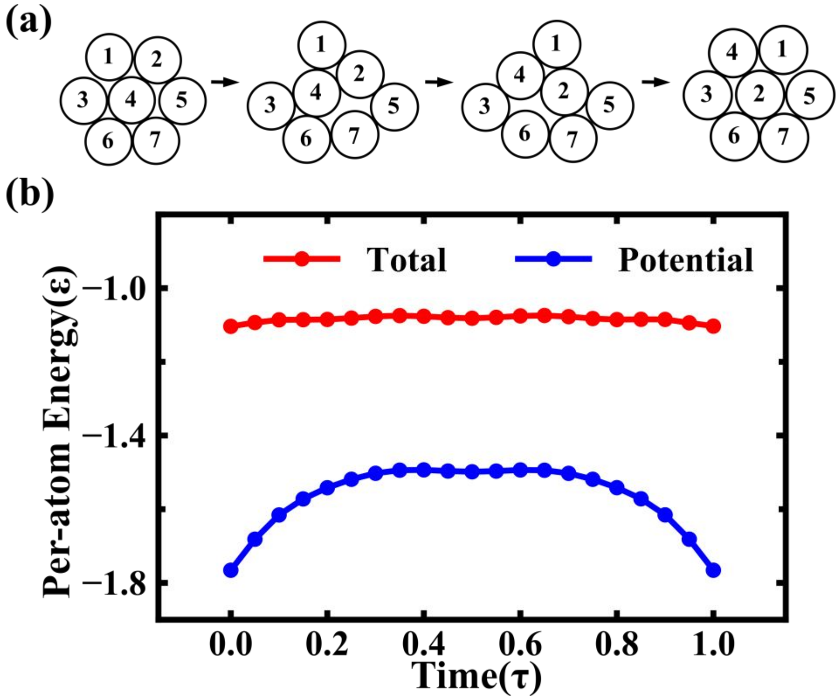


Figure S9: (a) transition path of a 2D cluster of 7 atoms for the migration of the center atom to the surface. In reduced LJ units, the transition takes place within 1 τ and the timestep is 0.01 τ. (b) Total energy (red) and potential energy per atom as a function of time.

**References**

(1) White, J. A. Lennard-Jones as a Model for Argon and Test of Extended Renormalization Group Calculations. *J. Chem. Phys.* **1999**, *111*, 9352–9356. DOI: 10.1063/1.479848.

(2) Allen, M. P.; Tildesley, D. J. *Computer Simulation of Liquids: Second Edition*, 2nd ed.; Oxford University Press: Oxford, 2017.

(3) Dellago, C.; Bolhuis, P. G.; Chandler, D. Efficient Transition Path Sampling: Application to Lennard-Jones Cluster Rearrangements. *J. Chem. Phys.* **1998**, *108*, 9236–9245. DOI: 10.1063/1.476378.

(4) Passerone, D.; Parrinello, M. Action-Derived Molecular Dynamics in the Study of Rare Events. *Phys. Rev. Lett.* **2001**, *87* , 108302. DOI: 10.1103/PhysRevLett.87.108302.
